# The Concept of Divergent Targeting through the Activation and Inhibition of Receptors as a Novel Chemotherapeutic Strategy: Signaling Responses to Strong DNA-Reactive Combinatorial Mimicries

**DOI:** 10.1155/2012/282050

**Published:** 2012-03-07

**Authors:** Heather L. Watt, Zakaria Rachid, Bertrand J. Jean-Claude

**Affiliations:** Cancer Drug Research Laboratory, Division of Oncology, Department of Medicine, McGill University Health Centre, Royal Victoria Hospital, Montreal, QC, Canada H3A 1A1

## Abstract

Recently, we reported the combination of multitargeted ErbB1 inhibitor–DNA damage combi-molecules with OCT in order to downregulate ErbB1 and activate SSTRs. Absence of translation to cell kill was believed to be partially due to insufficient ErbB1 blockage and DNA damage. In this study, we evaluated cell response to molecules that damage DNA more aggressively and induce stronger attenuation of ErbB1 phosphorylation. We used three cell lines expressing low levels (U87MG) or transfected to overexpress wildtype (U87/EGFR) or a variant (U87/EGFRvIII) of ErbB1. The results showed that Iressa ± HN2 and the combi-molecules, ZRBA4 and ZR2003, significantly blocked ErbB1 phosphorylation in U87MG cells. Addition of OCT significantly altered cell cycle distribution. Analysis of the DNA damage response pathway revealed strong upregulation of p53 by HN2 and the combi-molecules. Apoptosis was only induced by a 48 h exposure to HN2. All other treatments resulted in cell necrosis. This is in agreement with Akt-Bad pathway activation and survivin upregulation. Despite strong DNA damaging properties and downregulation of ErbB1 phosphorylation by these molecules, the strongest effect of SSTR activation was on cell cycle distribution. Therefore, any enhanced antiproliferative effects of combining ErbB1 inhibition with SSTR activation must be addressed in the context of cell cycle arrest.

## 1. Background

**Scheme 1:**
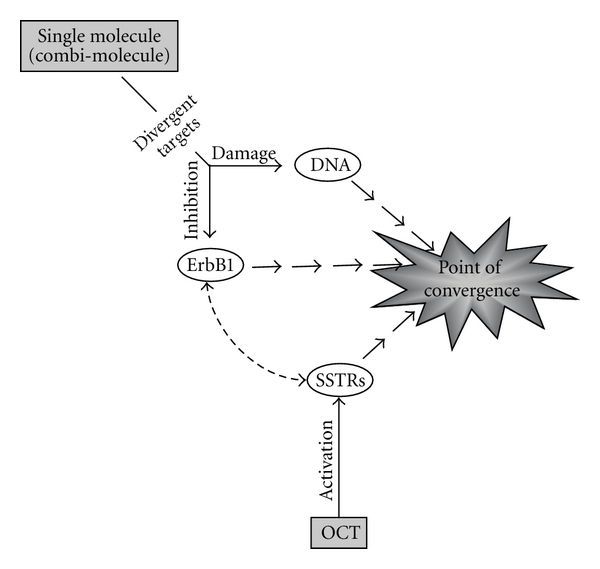
Principle of divergent targeting.

The genetic heterogeneity of solid tumours presents a challenge to cancer therapy such that single-targeted approaches, whether with nonselective cytotoxic drugs or highly specific kinase inhibitors, often fail due to the development of drug resistance. Invariably, as one receptor or pathway is blocked, alternate pathways substitute for the drug target. Moreover, if the target is not completely blocked, downstream components may be able to compensate. Therefore, modern chemotherapeutic strategies must adopt a more divergent targeting approach. Chemogenomic strategies seek to identify molecules which can target, upon minor modification, multiple members of the same family of proteins (e.g., protein kinases, GPCRs, or nuclear hormone receptors) [1, 2]. However, this remains a strategy whereby similar receptors with potentially similar functions within a tumour are targeted. The optimal strategy for an efficient multitargeting approach should be divergent to avoid the adverse effects of target redundancy at the advanced states of tumour progression. Over the past few years, we have designed molecules capable of targeting structurally unrelated cellular components (i.e., receptors and DNA). The fact that our unimolecular drugs that target both ErbB1 and DNA can be 10–20 times more potent than the combination of their single-target counterparts confirms the efficiency of divergent targeting [3–7]. Within the same context, we and others recently reported interactions between SSTRs (GPCRs) and ErbBs (RTKs) suggesting that these two receptor families might be ideal targets for our divergent strategy (Scheme 1) [8–10]. Therefore, we recently designed a divergent targeting strategy whereby we activated somatotstatin (SST) receptors (SSTRs) with octreotide (OCT), blocked epidermal growth factor (EGF) receptor (ErbB1/EGFR) with kinase inhibitors, and ErbB1-DNA targeting combi-molecules and induced DNA damage.

SST functions as a potent inhibitor of hormone and growth factor secretion as well as a modulator of cell proliferation through its cognate receptors SSTR1–5 and regulates a variety of signal transduction pathways including the mitogen-activated protein kinase (MAPK) pathway [11–16]. In contrast to SSTRs, ErbBs play fundamental roles in development, proliferation, differentiation, survival, and transformation [17–19]. Major ErbB1 downstream signalling pathways include Ras/Raf/MEK/MAPK, PI3K/Akt, STAT, and PLC*γ* [18, 20, 21]. While both SSTRs and ErbBs activate the MAPK pathway, SST-induced MAPK activation results in delayed cell cycle progression and EGF activation promotes proliferation. Therefore, SSTR and ErbB1 are true divergent targets.

In a recent study, we showed exacerbation of cell cycle perturbations following the combination of multitargeted ErbB1-DNA combi-molecules with OCT, a SSTR agonist [22]. The lack of translation into cell kill was believed to be in part due to insufficient ErbB1 inhibition and DNA damage. Here, we report the analysis of cell response following exposure to concurrent treatment of OCT with combinations of single-target molecules and unimolecular multitargeted combi-molecules that damage DNA more aggressively and induce stronger attenuation of ErbB1 phosphorylation.

**Scheme 2:**
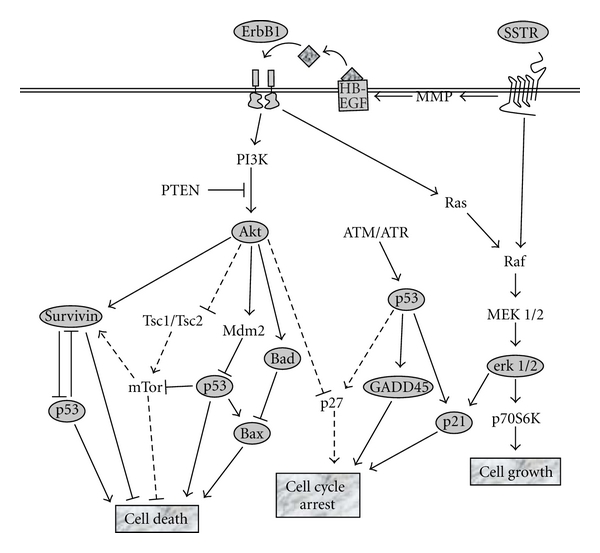
Key signalling pathways targeted with our divergent targeting strategy. The multitargeted approach includes activating SSTRs, inhibiting ErbB1, and inducing DNA damage. Key proteins analysed in this study are circled, while pathways not investigated are represented by dotted lines.

In this study, we combined strong ErbB1 TKIs with more potent chloroethylating DNA damaging drugs and investigated the cell signalling response to divergent targeting that induced concomitant ErbB1 inhibition, DNA damage, and SSTR activation. To this end, we analyzed the modulation of key proteins in the SSTR, MAPK, ErbB1-related signalling, and DNA damage response pathway (Scheme 2) as well as cell cycle distributions with the purpose of identifying a pharmacological effect (see point of convergence, Scheme 1) that is significantly enhanced by the divergent targeting process.

## 2. Methods

### 2.1. Materials

EGF was obtained from Roche Diagnostics (Indianapolis, IN). Mouse monoclonal antibodies against p21 (sc-817) and rabbit polyclonal antibodies against ErbB1 (sc-03), GADD45 (sc-797) and phosphotyrosine (sc-7020), were from Santa Cruz (Santa Cruz, CA). Rabbit polyclonal antibodies against phospho- and total Erk 1/2 (9101, 9102), phospho- and total p38 (9211, 9212), phospho- and total JNK (9251, 9252), phospho- and total p53 (9284, 2527), phospho- and total Akt (4060, 9272), and phospho- and total Bad (9291, 9295, 9292) were purchased from Cell Signalling Technology (Mississauga, ON). Rabbit polyclonal antibodies against survivin (AF886) were obtained from R&D Systems (Minneapolis, MN). Mouse monoclonal antibodies against XRCC1 (MS-434) and ERCC1 (MS-647) were purchased from LabVision (Fremont, CA). Ciprofloxacin and mouse monoclonal antibodies against *α*-actinin (A-5044) were from Sigma. Horseradish peroxidase-conjugated goat anti-rabbit and goat anti-mouse secondary antibodies (IgG) were from Jackson ImmunoResearch Laboratories (West Grove, PA). Cell culture media, Amphotericin B, HEPES, L-glutamine, and gentamycin sulfate were purchased from Wisent (St. Bruno, QC), while G418 was obtained from EMD Chemicals (Gibbstown, NJ). All other reagents were of analytical grade and purchased from various local suppliers.

### 2.2. Drug Treatment

**Figure 1:**
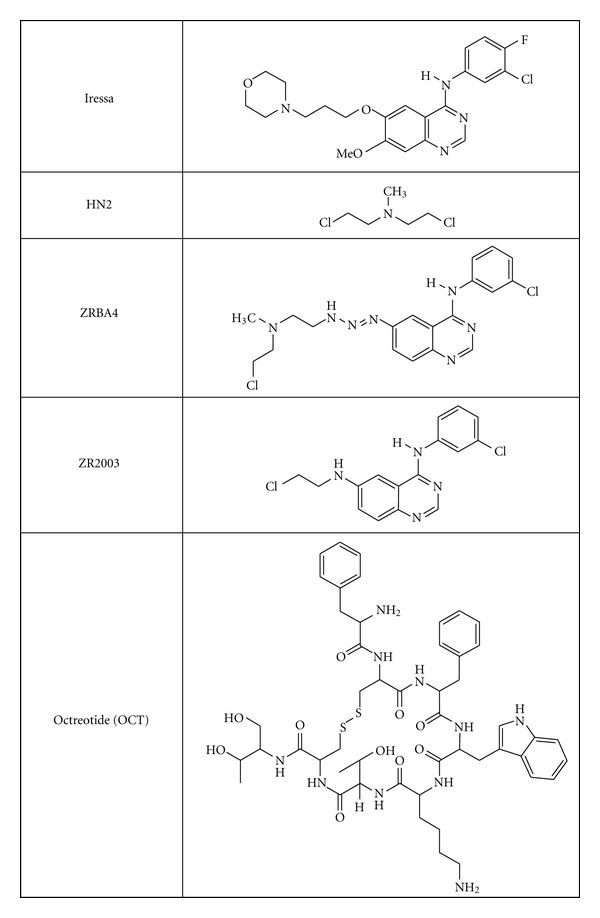
Chemical structures of Iressa, mechlorethamine (HN2), binary ErbB1-DNA damage combi-molecules (ZRBA4 and ZR2003), and octreotide (OCT).

ZRBA4 and ZR2003 were synthesized in our laboratory according to previously published procedures [23, 24]. Iressa (gefitinib) was provided by AstraZeneca, while mechlorethamine (HN2) was from Sigma and OCT was purchased from Bachem (Torrance, CA). The structures of these five drugs are presented in Figure 1. In all assays, drugs were resuspended in dimethyl sulfoxide (DMSO) and subsequently diluted in serum-supplemented medium immediately prior to use, unless otherwise specified. DMSO concentration never exceeded 0.2% (v/v).

We combined Iressa and HN2 at equimolar concentrations to maintain the same ratio of ErbB1 TKI: DNA damage molecules as the combi-molecules.

### 2.3. Cell Lines and Culture

U87MG glioma cells (American Type Culture Collection, Manassas, VA) and isogenic U87/EGFR and U87/EGFRvIII glioma cells (generous gifts from Dr. Frank Furnari, University of California, La Jolla, CA) were maintained in DMEM medium supplemented with 10% FBS, L-glutamine (1.76 *μ*M), HEPES (5.25 mg/mL), and antibiotics (26.8 *μ*M ciprofloxacin, 0.04 mg/mL gentamycin sulfate, 0.11 *μ*g/mL Amphotericin B) at 37°C in an atmosphere of 5% CO_2_/95% air. Selection pressure on the two transfected cell lines was maintained by supplementing the culture media with 400 *μ*g/mL G418. All experiments were performed on cells between passage 2 and 4. In all assays, cells were plated in DMEM without G418 24 h prior to treatment of subconfluent monolayers.

### 2.4. Alamar Blue Assay

Inhibition of cell proliferation was monitored with CellTiter Blue (Promega) as per the manufacturer's instructions. Briefly, cells were plated in 96-well plates and allowed to attach overnight. Cells were exposed to individual or combination treatments for six days. Treatments were terminated by the addition of 60 *μ*L CellTiter Blue (1 : 4 dilution in PBS). Plates were incubated at 37°C for an additional 2.5 h, while viable cells metabolized resazurin (maximum absorbance of 605 nm) into resorufin, a fluorescent metabolite (maximum absorbance of 573 nm). This translated into a fluorometric colour change that was captured using SOFTmax Pro 4.3LS (Molecular Devices, Sunnyvale, CA) connected to a SpectraMAX Gemini plate reader (Molecular Devices, Sunnyvale, CA). The following filters were used: 580 nm for excitation and 600 nm for fluorescence emission. Data were analysed using GraphPad Prism 4 (GraphPad Software Inc, San Diego, CA). IC_50_ values were calculated from three independent experiments run in triplicate. Statistical analysis was carried out using a one-way ANOVA, followed by a post hoc Tukey's test. *P* values < 0.05 were considered statistically significant.

### 2.5. Cell Cycle Analysis

Flow cytometric analysis of cell cycle profiles was performed on nonsynchronized cell populations as previously described with minor adjustments [25, 26]. Briefly, cells were plated in 6-well plates, allowed to grow until 65–75% confluency in serum-supplemented medium, and subsequently exposed to serial dilutions of drugs, alone or in combination, for 24 or 48 h at 37°C. Treatments were terminated by aspirating the media and rinsing the wells with PBS. Cells were subsequently collected by trypsinization, centrifuged (3500 rpm for 5 min) and washed twice with PBS. Cells were fixed by slowly adding 1 mL ethanol (70%) with continuous vortexing and then stored at 4°C for up to eight days. The day of analysis, cells were pelleted by centrifugation (3500 rpm for 5 min), rinsed twice with PBS and incubated with 200 *μ*L freshly prepared propidium iodide (PI)/RNAse solution (50 *μ*g/mL, 100 *μ*g/mL, resp.) in the dark for 30 min at 37°C. Data were collected using a BD FACSCalibur (BD Biosciences, Mississauga, ON) and the percentage of cells in each phase was calculated using FlowJo 8.4.6 (Tree Star, Ashland, OR). Data represent two independent experiments run in duplicate. Unpaired two-tailed *t*-test were used to identify significant changes in cell cycle distributions upon the addition of OCT. *P* values < 0.05 were considered statistically significant.

### 2.6. Apoptosis

Cell kill was determined by Annexin V-FITC binding as previously described with minor modifications [27]. Briefly, cells were plated in 6-well plates and allowed to attach overnight. Cells (65–75% confluent) were treated with a range of drug dilutions prepared in serum-supplemented medium for 24 or 48 h at 37°C. Treatments were terminated by rinsing the wells with PBS, and cells were collected by trypsinization followed by centrifugation (3500 rpm for 5 min). Cell pellets were washed twice with PBS and then resuspended in 1X binding buffer for a final concentration of 1 × 10^6^ cells/mL. Cells were treated with Annexin V-FITC and PI using the Apoptosis Detection Kit (BD Bioscience Pharmingen, San Jose, CA) according to the manufacturer's protocol. The reactions were subsequently quenched by the addition of 150 *μ*L 1X binding buffer. Data were collected using a BD FACSCalibur, and quadrant analysis of co-ordinate dot blots was performed using FlowJo 8.4.6. Data represent two independent experiments run in duplicate.

### 2.7. EGF-Induced Autophosphorylation Assay

U87MG cells were plated in 6-well plates using serum-supplemented medium and allowed to attach overnight. At 85–90% confluency, the wells were rinsed with PBS and the cells were starved for 24 h. Treatments consisted of 2 h exposures to the drugs followed by a quick rinse with PBS and a further 20 min treatment with 50 ng/mL EGF. Treatments were terminated by rinsing the wells with ice cold PBS and placing the plates on ice. Cells were scraped using a rubber policeman, and cell suspensions were transferred to labelled eppendorf tubes. Samples were centrifuged for two minutes at 10 000 rpm at 4°C, and the supernatant was removed. Cell pellets were homogenized in ice-cold lysis buffer (50 mM Tris-HCl pH 7.5, 150 nM NaCl, 1% NP-40, 1 mM EDTA, 5 mM NaF, 1 mM Na_3_VO_4_, 1 complete protease inhibitor tablet Roche Biochemicals, Laval, QC) and incubated for 30 min on ice. Samples were centrifuged at 10 000 rpm for 20 min at 4°C to remove cellular debris. Protein concentrations of the supernatants were determined by Bradford assay using known dilutions of BSA as standards. Samples (40 *μ*g) were solubilized in Laemmli sample buffer, placed in boiling water for 5 min and fractionated by electrophoresis on a 10% SDS-polyacrylamide gel. The fractionated proteins were transferred by electrophoresis to 0.2 *μ*m polyvinylidene difluoride (PVDF) membranes (Millipore, Bedford, MA). Membranes were blocked, incubated with primary antibodies and then HRP-conjugated secondary antibodies (1 : 25 000 in 0.1% TBST) followed by chemiluminescence detection with the ECL Advance Western Blotting Detection kit (Amersham Biosciences) in accordance with the manufacturer's instructions. Molecular weights were estimated using the BenchMark prestained Western Protein Standard (Invitrogen). Images were captured using an Alpha Innotech FluorChem 8800 gel box imager, and densitometry was carried out using FluorChem software (Alpha Innotech Co.). Percent changes in ErbB1 tyrosine phosphorylation expression were calculated as the ratio between the density of the phosphorylated tyrosine band (185 kDa) and the band density for EGFR. These values were subsequently corrected for loading (using *α*-actinin) and then for basal expression (control level was set at 1).

### 2.8. Western Blot Analysis

To investigate Erk 1/2 and Akt inhibition, U87 glioma cells were cultured and treated as described for the EGF-induced autophosphorylation assay. All other Western Blot analyses were executed on cells that were grown and treated in serum-supplemented medium for 48 h. Protein extraction and quantification were performed as described in the EGF-induced autophosphorylation assay. Samples (40 or 100 *μ*g) were solubilized in Laemmli sample buffer, boiled, and fractionated by electrophoresis on 10, 12, or 15% SDS-polyacrylamide gels. The fractionated proteins were transferred to PVDF membranes. The membranes were subsequently blocked, incubated with primary antibody and then with HRP-conjugated secondary antibody (1 : 25 000 dilution in 0.1% TBST). Signals were detected with the ECL Advance Western Blotting Detection kit in accordance with the manufacturer's instructions. Molecular weights were estimated using the BenchMark prestained Western Protein Standard. Images were captured using an Alpha Innotech FluorChem 8800 gel box imager, and densitometry was carried out using FluorChem software. Percent changes in protein activation (p-Erk 1/2, p-Akt, p-Bad) were calculated as the ratio between the density of the phosphorylated band and the band density for total Erk 1/2, Akt, or Bad, respectively. These values were subsequently corrected for loading (using *α*-actinin) and then for basal expression (control level was set at 1). Expression levels of all other proteins were calculated as the ratio between the density of the band of interest and the band density for the loading control (i.e., *α*-actinin). These values were subsequently corrected for basal expression (control level was set as 1).

## 3. Results

### 3.1. Inhibition of ErbB1-Mediated Signalling

**Figure 2:**
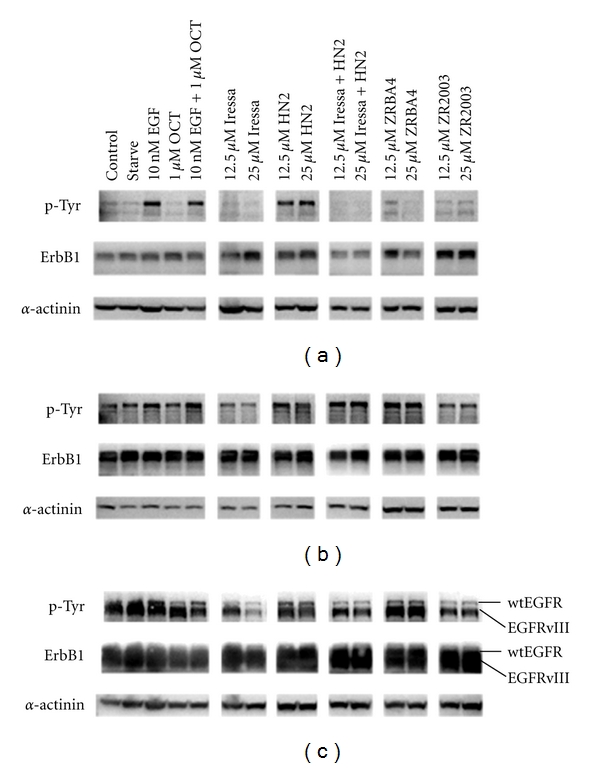
Dose-dependent inhibition of EGF-induced ErbB1 activation in U87MG, (a) U87/EGFR (b), and U87/EGFRvIII (c) glioma cells. Serum-starved cells were treated with the indicated concentrations of Iressa ± mechlorethamine (HN2), ZRBA4, or ZR2003 for 2 h followed by a 20 min EGF (50 ng/mL) stimulation. Cell lysates (40 *μ*g) were fractionated by SDS-PAGE and probed with antiphosphotyrosine (1 : 1000) antibodies (see Materials and Methods for details). Blots were subsequently stripped and reprobed for total ErbB1 (1:1000) followed by *α*-actinin (1 : 1500). Major protein bands of 170 (ErbB1) and 100 kDa (*α*-actinin) were obtained.

Recent studies have reported strong ErbB1 TK inhibitory activity for ZRBA4 (4.4 nM) and ZR2003 (26 nM) [23, 24]. Therefore, we first investigated their ability to inhibit ErbB1 activation in our isogenic panel of brain tumour cells. All ErbB1 TKIs (Iressa, ZRBA4 and ZR2003) attenuated EGF-induced ErbB1 tyrosine phosphorylation in U87MG as well as U87/EGFRvIII cells (Figure 2). Moreover, Iressa and ZR2003 slightly decreased EGFRvIII phosphorylation. Similarly, at the concentrations tested, only Iressa and ZR2003 blocked ErbB1 phosphorylation in U87/EGFR cells.

**Figure 3:**
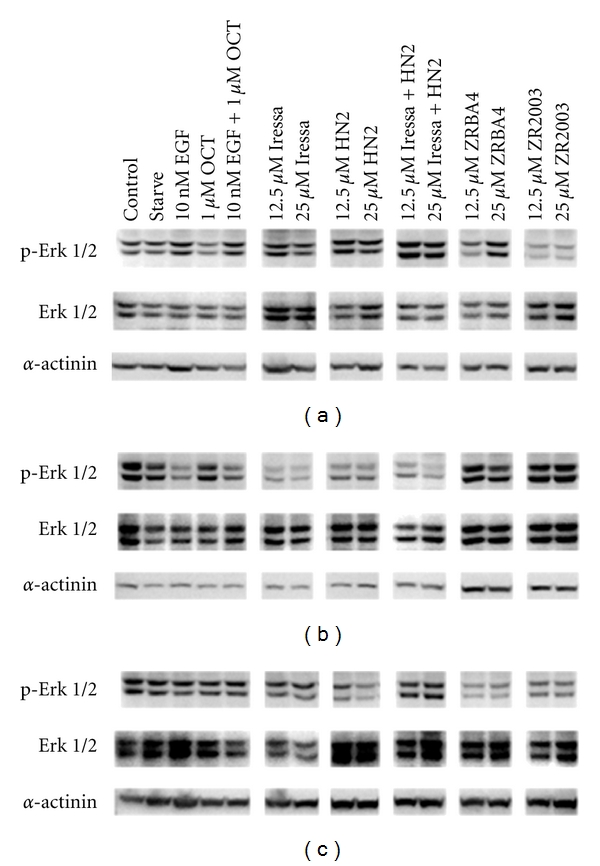
Dose-dependent inhibition of EGF-induced Erk 1/2 activation in U87MG (a), U87/EGFR (b) and U87/EGFRvIII (c) glioma cells. Serum-starved cells were treated with the indicated concentrations of Iressa ± mechlorethamine (HN2), ZRBA4 or ZR2003 for 2 h followed by a 20 min EGF (50 ng/mL) stimulation. Cell lysates (40 *μ*g) were fractionated by SDS-PAGE and probed with anti-phospho-Erk 1/2 (1 : 1000) antibodies (see Materials and Methods for details). Blots were subsequently stripped and reprobed for total Erk 1/2 (1 :1000) followed by *α*-actinin (1:1500). Major protein bands of 44, 42 (Erk 1/2) and 100 kDa (*α*-actinin) were obtained.

We next determined whether inhibition of ErbB1 phosphorylation translated into attenuation of the downstream MAPK pathway, through which EGF induces proliferation (Figure 3). Iressa and ZR2003 inhibited Erk 1/2 phosphorylation in U87MG cells, while only Iressa attenuated Erk 1/2 activation in U87/EGFR cells. While ErbB1 phosphorylation in the U87/EGFRvIII cell line was inhibited by all TKIs or combi-molecules, they only induced moderate inhibition of EGFRvIII phosphorylation. Therefore, we had in hand all the levels of effects needed to examine cell response to the divergent targeting approach. When the combi-molecules or combination of Iressa + HN2 were coadministered with the SSTR agonist octreotide (OCT), no significant change in Erk1/2 phosphorylation status of the cells was observed (data not shown). Total Erk 1/2 was relatively even across all treatments in U87 and U87/EGFR cell lines, while a dose-dependent increase was observed in U87/EGFRvIII calls, an effect that may be specific to the latter cell type.

### 3.2. Cell Cycle Analysis

#### 3.2.1. HN2 + Iressa ± OCT-Induced Cell Cycle Perturbations

**Figure 4:**
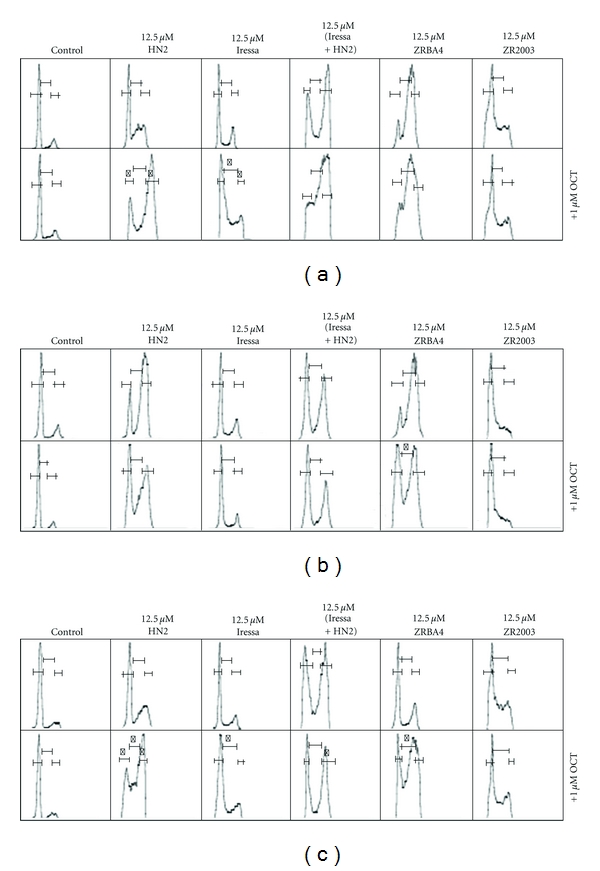
Representative histograms illustrating Iressa, mechlorethamine (HN2), Iressa + HN2, ZRBA4, and ZR2003-induced cell cycle arrest in U87MG (a), U87/EGFR (b), and U87/EGFRvIII (c) glioma cells following a 48 h treatment. Cell cycle perturbations following concomitant treatment with 1 *μ*M octreotide (OCT) are shown in the lower panels. *Shows statistical differences, within the same phase of the cell cycle, between drug alone and drug + OCT (*P* < 0.05).

HN2 is a bifunctional alkylating agent that induces high levels of DNA cross-links. It is known to induce cell cycle arrest at all phases of the cell cycle. At high concentrations, it blocks cells in G1 and at low concentrations, it induces cell cycle arrest in S and G2/M. On the other hand, Iressa is known to arrest cells in G1. As demonstrated in Figure 4 and Table 1, treatment with 12.5 *μ*M of HN2 induced cell cycle arrest in S in U87MG and U87/EGFRvIII transfectant cells but strong S (late) and G2/M arrest in the U87/EGFR cells. Surprisingly, Iressa induced some cell cycle arrest in the S phase. When the two drugs (HN2 and Iressa) were combined, a dramatic change in cell cycle distribution was induced leading to increased S (late) and G2/M arrest in all three cell types. More importantly, addition of OCT shifted the cell cycle arrests to S.

#### 3.2.2. ZRBA4 ± OCT-Induced Cell Cycle Perturbations

ZRBA4 is designed to be a prodrug of a DNA cross-linking alkylating species similar to HN2 and an ErbB1 TKI. It is therefore a unimolecular mimic of the HN2 + Iressa combination. ZRBA4 induced cell cycle arrest in S and G2M in U87MG and U87/EGFR cells (Figure 4, Table 1). Addition of OCT further perturbed cell cycle distribution profiles in a cell-dependent manner. U87MG cells shifted from S and G2/M arrest to the S phase. In contrast, OCT enhanced the accumulation of U87/EGFR cells in G1 at the expense of the S phase while leaving the G2/M population unchanged. Meanwhile, U87/EGFRvIII cells accumulated in the G2/M phase of the cell cycle in the absence of OCT. However, addition of OCT dramatically changed the cell cycle profile, leading to strong cell cycle arrest in late S and G2M.

#### 3.2.3. ZR2003 ± OCT-Induced Cell Cycle Perturbations

ZR2003 is a combi-molecule that does not require metabolic activation to generate its DNA damaging species: it can either block ErbB1 tyrosine kinase activity or damage DNA, and, unlike ZRBA4 and HN2, it cannot generate DNA cross-links. Therefore, its mechanism of action is different from that of ZRBA4. Interestingly, while ZR2003 induced S phase arrest in all three isogenic cell lines (Figure 4, Table 1), its effect was not altered by OCT, indicating the effects of OCT may be dependent on the type of DNA lesions induced by these drugs.

### 3.3. p53 Expression and Ser15 Phosphorylation

**Figure 5:**
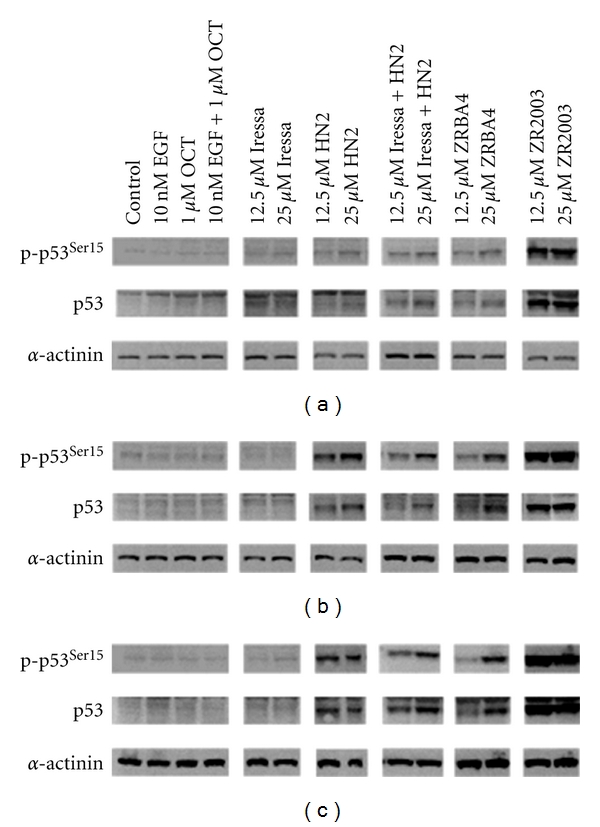
Dose-dependent changes in p53 phosphorylation (Ser15) and expression in U87MG (a), U87/EGFR (b), and U87/EGFRvIII (c) glioma cells treated for 48 h. Cell lysates (40 *μ*g) were fractionated by SDS-PAGE and probed with antiphospho-p53 (1 : 1000) antibodies (see Materials and Methods for details). Blots were subsequently stripped and reprobed for total p53 (1 : 1000) followed by *α*-actinin (1 : 1500). Major protein bands of 53 (p53) and 100 kDa (*α*-actinin) were obtained.

Upon DNA damage, Ataxia-telangiectasia (ATM), ATM and Rad3-related (ATR), and DNA-dependent protein kinase (DNA-PK) activate p53 through phosphorylation at Ser15 [28, 29]. We detected a dose-dependent phosphorylation of p53 at Ser15 in all samples treated with HN2, alone or in combination with Iressa, and ZRBA4 (Figure 5). Moreover, treatment with HN2, HN2 + Iressa, and ZRBA4 enhanced p53 accumulation. Meanwhile, ZR2003, a type II combi-molecule, elicited the greatest activation as well as accumulation of p53 in all three cell lines. Finally, combination of OCT with these treatments did not enhance p53 activation nor expression.

### 3.4. Alterations of Key Players in the Cell Cycle

To elucidate the pathway through which SSTR activation could enhance HN2 ± Iressa and ZRBA4-induced cell cycle arrest, we investigated changes in p21, a signalling intermediate for SSTRs as well as other pathways that play a role in cell cycle arrest. Since OCT enhanced HN2- and ZRBA4-induced S and G2/M arrest, we investigated whether this effect was mediated by p21. Unfortunately, p21 was not detectable in these cells, potentially due to downregulation by Akt. Based on these results, we decided to verify the expression of GADD45, another signalling intermediate in p53-induced G2/M arrest. The results showed that GADD45 was activated wherever p53 was phosphorylated (data not shown).

### 3.5. Effect of ErbB1 Inhibition on DNA Repair Proteins

**Figure 6:**
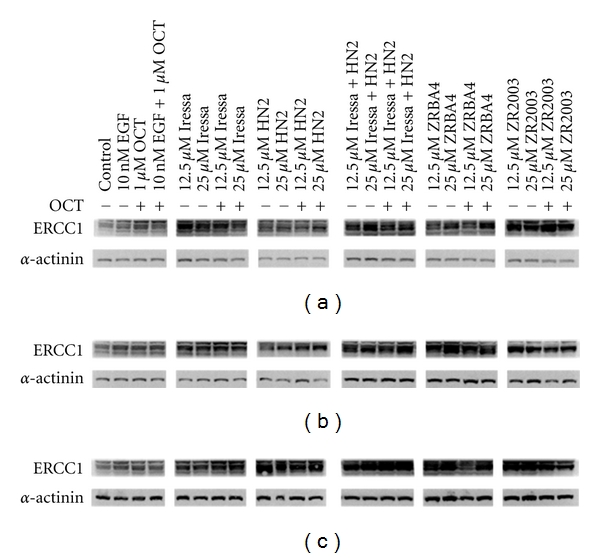
Upregulation of ERCC1 expression in U87MG (a), U87/EGFR (b), and U87/EGFRvIII (c) glioma cells. Cells were treated with the indicated concentrations of Iressa, mechlorethamine (HN2), Iressa + HN2, ZRBA4, or ZR2003, alone or in combination with octreotide (OCT) for 48 h. Cell lysates (40 *μ*g) were fractionated by SDS-PAGE and probed with anti-ERCC1 (1 : 1000) antibodies (see Materials and Methods for details). Blots were subsequently stripped and reprobed for *α*-actinin (1 : 1500). Major protein bands of 36 (ERCC1) and 100 kDa (*α*-actinin) were obtained.

Eukaryotes have developed multiple types of DNA repair systems to ensure genomic fidelity before replication. ATM, ATR, and DNA-PK kinases check genomic integrity at the G1/S and G2/M checkpoints. Moreover, stimulation of ErbB1 has been reported to induce DNA repair proteins such as ERCC1 and XRCC1 [30]. The former plays a role in nucleotide excision repair (NER) and recombination repair, while the latter is involved in base excision repair (BER) and nonhomologous end-joining (NHEJ). HN2, as a bifunctional alkylator, damages DNA by alkylating its bases mainly at the N7 position of guanine [31–33]. This can result in DNA base pair mismatches as well as intra- and interstrand crosslinks. The N7-alkyl guanine can be repaired by BER, while the crosslinks are generally repaired by homologous recombination repair (HRR) and NHEJ. ERCC1 was upregulated in all three cell lines following a 48 h exposure to Iressa + HN2, ZRBA4, or ZR2003 (Figure 6). ZR2003 elicited the strongest response in U87MG cells, while ERCC1 expression in U87/EGFR cells was most strongly upregulated in response to ZRBA4. Moreover, U87/EGFRvIII cells showed the strongest upregulation of ERCC1 with Iressa + HN2, ZRBA4, and ZR2003 eliciting similar degrees of upregulation. In contrast, XRCC1 was not detected over the course of the 48 h treatments (data not shown).

### 3.6. Antiproliferative Activity of ZRBA4, ZR2003, Iressa, HN2, and OCT

**Figure 7:**
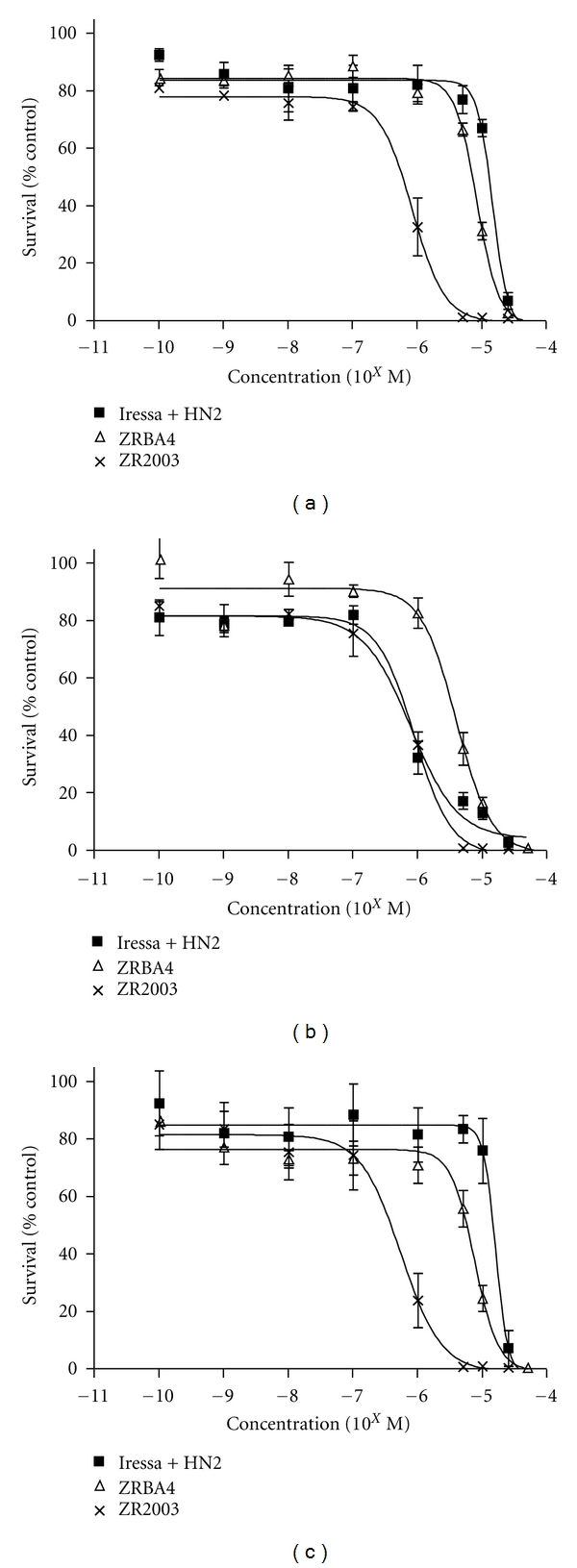
Relative growth inhibition of ZRBA4, ZR2003, and equimolar combination of Iressa + mechlorethamine (HN2) in U87MG (a), U87/EGFR (b), and U87/EGFRvIII (c) isogenic glioma cells. Cells were exposed to each drug for 6 days, and growth inhibition was measured by alamar blue assay (see Materials and Methods for details). Each point represents three independent experiments run in triplicate.

We next investigated the anti-proliferative effects of ZRBA4, ZR2003, Iressa, and HN2, alone as well as in combination with OCT, using a 6-day alamar blue assay (Table 2). ZRBA4, a type I combi-molecule, demonstrated 1.4-and-4.5 fold superior antiproliferative activity (*P* < 0.05) over Iressa in U87MG and U87/EGFRvIII cells, respectively. ZRBA4 also slightly enhanced growth inhibition over HN2 in U87MG cells, while it induced a 2.6-fold increase in cell kill compared with HN2 in U87/EGFRvIII cells. Consistent with the combi-targeting concept, a 6-day treatment with ZRBA4 resulted in a 1.8-to 2.1-fold superior inhibition of proliferation (*P* < 0.05) compared with the two-drug Iressa + HN2 combination in U87MG and U87/EGFRvIII cells (Figure 7, Table 2). In addition, ZRBA4 showed 2.1-fold selectivity for ErbB1-overexpressing cells (*P* < 0.05); however, in U87/EGFR cells, it was less effective at inhibiting proliferation than Iressa, HN2, and Iressa + HN2. In contrast, ZR2003, a type II combi-molecule, demonstrated a slight selectivity (1.5-fold) for the EGFRvIII mutation. It showed 14.5-, 3.4-, and 63-fold superior antiproliferative activity (*P* < 0.05) over Iressa in U87MG, U87/EGFR, and U87/EGFRvIII cells, respectively. Moreover, in U87/EGFRvIII cells, ZR2003 demonstrated a statistically significant (*P* < 0.05) 29.3-fold enhancement of cell kill over Iressa + HN2 (Figure 7). A more moderate 11.7-and 17.7-fold increase in growth inhibition (*P* < 0.05) was detected in U87MG cells treated with HN2 or Iressa + HN2, respectively. We also investigated whether simultaneous activation of SSTRs with OCT would enhance the antiproliferative activity of the binary-targeted combi-molecules but did not detect any significant interactions.

### 3.7. Apoptosis

**Figure 8:**
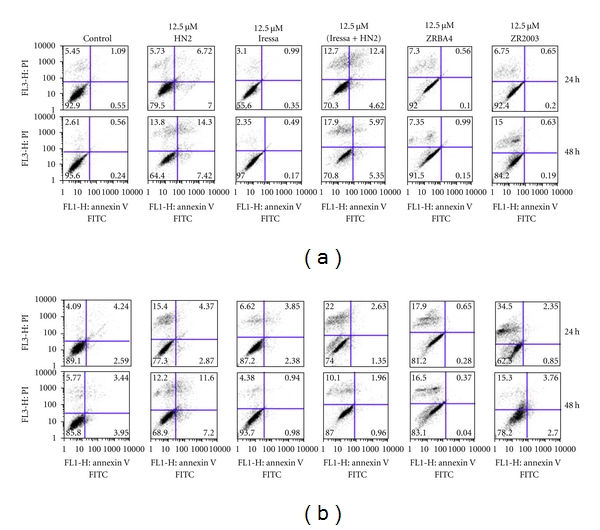
Representative Annexin V/propidium iodide (PI) intensity dot blots of U87MG (a) and U87/EGFR (b) cells treated for 24 (upper panels) or 48 h (lower panels) with Iressa, mechlorethamine (HN2), Iressa + HN2, ZRBA4, or ZR2003. Cell death was determined by Annexin V and propidium iodide (PI) staining (see Materials and Methods for details). Data are the mean of two independent experiments run in duplicate.

**Figure 9:**
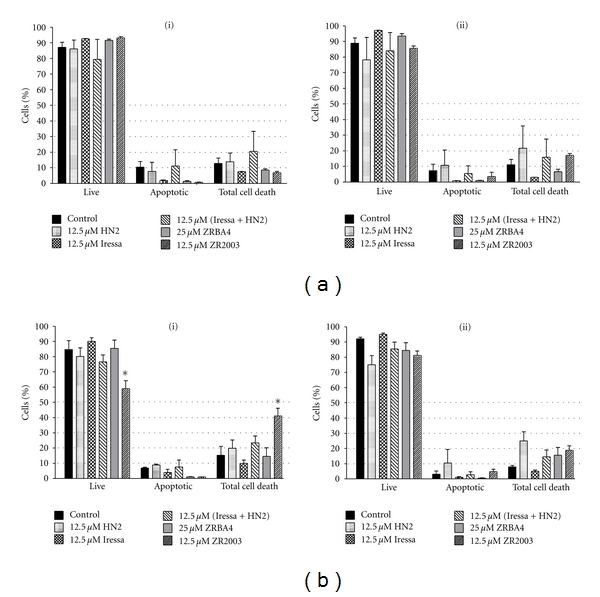
Assessment of apoptotic effects induced by Iressa ± mechlorethamine (HN2), ZRBA4, and ZR2003. U87MG (a) and U87/EGFR (b) cells were treated for 24 (i) or 48 h (ii). Levels of cell death were determined by Annexin V and propidium iodide (PI) staining. Bars for apoptotic cell death represent the mean percentage of Annexin-V-positive cells. Total cell death encompasses early and late apoptotic as well as necrotic cell death. Data are the mean of two independent experiments run in duplicate. *Statistically different from control (*P* < 0.05).

We subsequently determined how the observed cell cycle perturbations would translate into apoptosis (Figures 8 and 9). Cell death was induced in the three cell lines by HN2 as well as Iressa + HN2. Interestingly, a shorter (24 h) treatment with HN2 mainly induced a nonapoptotic cell death pathway, while we detected some cells undergoing apoptosis following a longer (48 h) exposure (Figure 8). Meanwhile, ZRBA4 induced minimal cell death in U87/EGFRvIII (data not shown) and U87MG cells and nonapoptotic cell death in U87/EGFR cells (Figure 8). Moreover, ZR2003 showed dose-dependent induction of cell death with relatively strong early (within 24 h) induction of nonapoptotic cell death (Figure 8). Finally, when we combined 1 *μ*M OCT with the above treatments, we did not detect any potentiation of cell death.

### 3.8. Modulation of Apoptotic as well as Antiapoptotic Signalling

To rationalize the lack of apoptosis, we extended our investigation to the analysis of key components of the DNA damage response pathway and the intrinsic apoptotic pathway. Based upon reports that DNA alkylators, including HN2, induce apoptosis partially through JNK activation, we investigated JNK and p38 activation by Western Blot analysis [34, 35]. Neither JNK nor p38 activation were detected (data not shown). This is consistent with the lack of induced apoptosis.

The PI3K/Akt pathway, another major downstream effector pathway of ErbB1, promotes cell survival by inhibiting apoptosis as well as modulating cell cycle arrest. We, therefore, verified whether the combi-molecules could alter Akt phosphorylation. However, due to a PTEN mutation in these cells, Akt was constitutively phosphorylated at Ser473 and unresponsive to ErbB1 inhibition (data not shown).

Since these cells responded to DNA damage with p53 activation as well as upregulation, we further extended our investigation and verified Bad phosphorylation at both Ser112 and Ser136. Phosphorylation of these two sites (by MEK1 and Akt, respectively) plays a critical role in cell survival through sequestration of Bad with 14-3-3 proteins thereby preventing Bad from binding Bcl-2 or Bcl-xL and subsequently releasing proapoptotic Bax. Therefore, we determined the extent to which our drugs modulated Bad phosphorylation. We also investigated whether combining OCT with the above treatments would further alter Bad phosphorylation. Bad was constitutively phosphorylated at Ser112 and Ser136 in U87 and U87/EGFRvIII cells (data not shown). In contrast, total Bad was barely detectable in U87/EGFR cells (data not shown). No treatment reduced Bad phosphorylation at either site. Moreover, OCT had no effect on Bad phosphorylation (data not shown).

**Figure 10:**
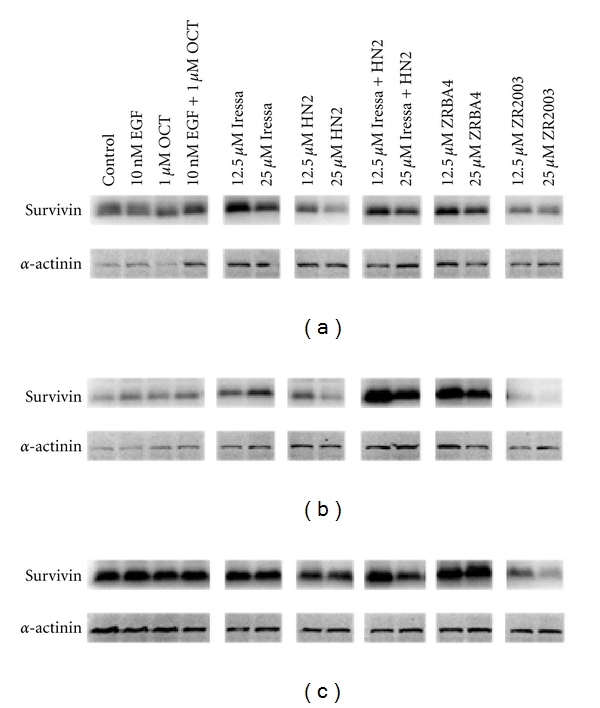
Treatment-dependent regulation of survivin expression in U87MG (a), U87/EGFR (b), and U87/EGFRvIII (c) glioma cells. Cells were treated with the indicated concentrations of Iressa, mechlorethamine (HN2), Iressa + HN2, ZRBA4 or ZR2003 for 48 h. Cell lysates (40 *μ*g) were fractionated by SDS-PAGE and probed with antisurvivin (1 : 2000) antibodies (see Materials and methods for details). Blots were subsequently stripped and reprobed for *α*-actinin (1 : 1500). Major protein bands of 16 (survivin) and 100 kDa (*α*-actinin) were obtained.

SSTR3 has been shown to play a role in p53-mediated apoptosis, while the other four SSTRs induce cell cycle arrest via p21 or p27. With no clear enhancement of p53, Bad, p21, or GADD45 by OCT, we extended our study to include another key protein in apoptosis, survivin. Survivin, an inhibitor of apoptosis protein (IAP), is most recognized for its role in chromosome segregation and cytokinesis [36, 37]. In addition to its role in cell division, survivin overexpression is associated with inhibition of apoptosis via both the extrinsic as well as intrinsic pathways although it is more efficient at blocking the latter pathway [38, 39]. In general, survivin and p53 negatively regulate each other's expression. However, as illustrated in Figure 10, ZRBA4 enhanced survivin as well as p53 (Figure 5) expression confirming blockage of apoptosis. In contrast, in HN2- and ZR2003-treated gliomas cells, p53 and survivin were inversely related (Figures 5 and 10) while Iressa attenuated the HN2-mediated inhibition of survivin. We did not detect any OCT-induced regulation of survivin expression (data not shown).

## 4. Discussion

The effectiveness of single-targeted cancer therapies is mitigated by the inevitable onset of drug resistance. This may arise due to alternate pathways compensating for the drug target or to the accumulation of mutations within the target or components of downstream signalling pathways. Therefore, classical cancer therapies generally combine multiple drugs with different mechanisms of action to prevent drug resistance. However, due to the nonspecific nature of their binding, some combinations can result in increased toxicity. Moreover, the potency of these drugs is often mitigated by DNA repair pathways. Therefore, novel chemotherapeutic approaches are urgently needed. In this study, we examined our divergent targeting strategy using DNA damage, ErbB1 TK inhibition, and SSTR activation. We used OCT as our SSTR agonist, while HN2 + Iressa, ZRBA4 and ZR2003, chloroethylating combi-molecules, induced concurrent DNA damage and ErbB1 inhibition.

Previous studies have demonstrated the binary ErbB1-DNA targeting properties of ZRBA4 as well as its ability to induce DNA interstrand crosslinks in a manner similar to that of HN2 [23]. Consistent with literature, ZRBA4, as a partially irreversible ErbB1 inhibitor, showed selectivity for ErbB1-overexpressing cells. Furthermore, it manifested characteristics of both components (DNA damage and ErbB1 inhibition) as outlined by the similarity of the cell cycle perturbation profile that it induced when compared with equivalent two-drug combinations of HN2 + Iressa. Likewise, cell cycle arrest induced by the two forms of combinations (i.e., individual drugs or unimolecular combi-molecules) was significantly enhanced by OCT activation of SSTRs in these cells. Notably, OCT altered the cell cycle distribution profile of cells exposed to the DNA damaging agent HN2 more dramatically than those treated with the ErbB1 inhibitor Iressa. However, its effect was more dramatic when HN2 was combined with the ErbB1 inhibitor, suggesting that ErbB1 inhibition plays a role in the overall cell cycle perturbation. As outlined in Scheme 2, SSTR activation induces cell cycle inhibitors, while ErbB1 phosphorylation downregulates them. Thus, inhibition of ErbB1 that leads to downregulation of downstream signalling (e.g., Erk1/2 activation) may relieve any antagonistic effect to SSTR-mediated cell cycle arrest.

 In general, SSTR1, 2, 4, and 5 induce G1 arrest in a p53-independent manner while SSTR3 induces apoptosis through a p53/Bax-dependent mechanism [40–43]. Previous studies have shown that SSTR3-mediated p53 activation occurs independently of cell cycle arrest and p21 induction [44]. Moreover, p53-mediated activation of p21 promotes G1 arrest while loss of G1/S checkpoint control generally sensitizes cells to DNA damage. We did not detect p21 in our samples nor did the nitrogen mustard-containing compounds (HN2, ZRBA4, and ZR2003) induce significant G1 arrest, suggesting that the p53 activation and upregulation observed in the Western Blot analyses may not be related to cell cycle arrest. Thus the increased effect conferred by OCT may be due to a direct effect of SSTR activation on the cell cycle through induction of cell cycle inhibitors or other proteins that trigger the cells to arrest earlier in the cycle in response to the DNA damage induced by HN2 and our combi-molecules.

While p53 is generally associated with the induction of apoptosis, the absence of Bax upregulation and Bad dephosphorylation demonstrates that the p53-mediated apoptotic pathway is blocked. Moreover, the increased expression of survivin, an inhibitor of apoptosis protein (IAP), provides further evidence that the drugs tested in this study cannot induce apoptosis. However, HN2 (but not Iressa + HN2) activated and upregulated p53 as well as downregulated survivin expression after a 48 h treatment which translated into cells dying by both apoptosis (ca. 50%) and a nonapoptotic form of cell death. In contrast, ZR2003 showed the strongest p53 activation of all the drugs and combinations of drugs tested in this study. Yet, the concomitant decrease in survivin levels did not translate into increased apoptosis suggesting that the observed decline was not due to drug-induced inhibition, as observed with HN2, but was perhaps due to rapid turnover (*t*1/2 = 30 min) of the protein in the G1 phase of the cell cycle [45]. *In toto*, these data suggest that the concomitant inhibition of survivin may allow these cells to undergo apoptosis.

## 5. Conclusions

In summary, the results presented herein demonstrate that ErbB1 TKIs inhibit ErbB1 but not EGFRvIII phosphorylation. Moreover, due to a PTEN mutation, Akt was constitutively active and Bad remained phosphorylated, preventing cells from undergoing apoptosis upon ErbB1 inhibition. This may have played a role in p21 downregulation which could explain the absence of OCT-induced cell cycle arrest on its own. However, OCT potentiated arrest in the S-phase of the cell cycle when combined with Iressa ± HN2, or ZRBA4. Moreover, both Bax unresponsiveness to p53 activation and survivin upregulation despite p53 activation may contribute to the cells dying via a nonapoptotic pathway. Thus, future studies to improve the divergent targeting strategy should be directed at bridging the strong cell cycle perturbation observed to a cell death pathway.

##  Conflict of Interests

The authors declare that they have no competing interests.

##  Authors' Contributions

Dr. H. L. Watt performed the experimental work and prepared the paper. Dr. Z. Rachid synthesized ZRBA4 and ZR2003. Dr. B. Jean-Claude revised the paper.

## Figures and Tables

**Table 1:** Relative distribution of U87MG, U87/EGFR, and U87/EGFRvIII glioma cells across the G1, S, and G2 phases of the cell cycle. Cells were treated for 48 h with Iressa, mechlorethamine (HN2), Iressa + HN2, ZRBA4, and ZR2003 in the absence or presence of 1 *μ*M octreotide (OCT).

		U87MG	U87/EGFR	U87/EGFRvIII
Control	G1	73.0 ± 3.4	80.0 ± 2.2	83.3 ± 0.2
	S	10.2 ± 1.5	4.2 ± 2.1	7.6 ± 0.6
	G2	17.3 ± 1.6	15.7 ± 0.1	9.1 ± 0.3

1 *μ*M OCT	G1	77.2 ± 0.8	82.7 ± 2.9	82.7 ± 1.2
	S	9.3 ± 0.3	5.9 ± 3.0	7.0 ± 1.1
	G2	18.0 ± 2.6	11.5 ± 0.1	10.3 ± 0.7

12.5 *μ*M HN2	G1	51.5 ± 7.8	20.7 ± 2.7	55.0 ± 0.2
	S	36.1 ± 1.7	49.1 ± 8.6	24.8 ± 1.8
	G2	20.1 ± 0.7	45.5 ± 0.9	20.1 ± 1.7

12.5 *μ*M HN2 + 1 *μ*M OCT	G1	18.7 ± 2.2*	30.6 ± 3.8	19.6 ± 4.7*
	S	48.7 ± 7.5	28.9 ± 2.7	71.7 ± 2.2*
	G2	59.5 ± 2.3*	35.0 ± 2.4	41.8 ± 1.8*

12.5 *μ*M Iressa	G1	49.6 ± 5.7	63.6 ± 0.4	58.2 ± 2.3
	S	18.6 ± 1.2	15.3 ± 2.3	15.7 ± 0.5
	G2	21.9 ± 1.0	22.1 ± 2.9	22.5 ± 1.2

12.5 *μ*M Iressa + 1 *μ*M OCT	G1	53.1 ± 4.7	58.3 ± 2.5	66.3 ± 2.8
	S	40.5 ± 2.6*	22.7 ± 2.1	24.7 ± 2.3*
	G2	16.6 ± 1.7*	16.0 ± 1.7	13.6 ± 1.4

12.5 *μ*M (Iressa + HN2)	G1	25.7 ± 6.4	32.0 ± 3.4	32.5 ± 9.3
	S	19.7 ± 1.8	32.0 ± 5.9	23.5 ± 9.5
	G2	39.6 ± 17.4	33.6 ± 1.8	44.7 ± 0.3

12.5 *μ*M (Iressa + HN2) + 1 *μ*M OCT	G1	23.5 ± 3.9	39.5 ± 3.8	34.9 ± 3.9
	S	51.9 ± 10.1	18.9 ± 2.4	17.2 ± 5.3
	G2	19.0 ± 2.9	22.3 ± 2.2	52.1 ± 1.2*

12.5 *μ*M ZRBA4	G1	12.2 ± 0.3	15.6 ± 0.2	60.5 ± 0.3
	S	56.1 ± 1.3	71.4 ± 1.8	12.9 ± 0.3
	G2	31.1 ± 0.8	33.5 ± 2.5	26.5 ± 0.1

12.5 *μ*M ZRBA4 + 1 *μ*M OCT	G1	20.3 ± 6.9	31.4 ± 2.3	22.9 ± 4.7
	S	59.3 ± 4.0	39.0 ± 4.1*	50.6 ± 3.2*
	G2	23.7 ± 3.4	28.8 ± 2.1	28.3 ± 0.8

12.5 *μ*M ZR2003	G1	52.1 ± 4.6	28.3 ± 1.9	35.3 ± 1.5
	S	26.3 ± 2.3	54.3 ± 2.7	53.5 ± 0.9
	G2	15.7 ± 2.0	12.7 ± 0.2	13.9 ± 1.7

12.5 *μ*M ZR2003 + 1 *μ*M OCT	G1	49.5 ± 4.6	32.6 ± 3.6	28.7 ± 1.9
	S	25.7 ± 1.6	57.8 ± 0.9	57.1 ± 0.5
	G2	19.3 ± 1.5	12.5 ± 1.3	12.1 ± 2.6

*Shows statistical differences, within the same phase of the cell cycle, between drug alone and drug + OCT (*P* < 0.05).

**Table 2:** Inhibition of U87MG, U87/EGFR, and U87/EGFRvIII cell growth by Iressa ± mechlorethamine (HN2), ZRBA4, and ZR2003, alone or in combination with 1 *μ*M octreotide (OCT), as assessed by the alamar blue assay.

	U87	U87/EGFR	U87/EGFRvIII
	IC_50_ (*μ*M)	IC_50_ (*μ*M)	IC_50_ (*μ*M)
OCT	n/a	n/a	n/a

Iressa	11.89 ± 8.40	2.96 ± 0.05	34.14 ± 3.53
Iressa + OCT	13.01 ± 9.70	3.27 ± 0.28	32.23 ± 3.80

HN2	9.58 ± 1.20	1.52 ± 0.59	19.74 ± 2.85
HN2 + OCT	n/a	1.81 ± 0.68	13.33 ± 3.78

Iressa + HN2	14.51 ± 0.20	0.77 ± 0.27	15.82 ± 1.97
Iressa + HN2 + OCT	12.24 ± 1.53	0.66 ± 0.13	n/a

ZRBA4	8.27 ± 0.57	3.91 ± 0.62	7.50 ± 0.82
ZRBA4 + OCT	8.20 ± 0.59	3.58 ± 0.52	7.45 ± 0.62

ZR2003	0.82 ± 0.24	0.87 ± 0.14	0.54 ± 0.18
ZR2003 + OCT	0.77 ± 0.19	0.73 ± 0.08	0.43 ± 0.14

Values are presented as mean ± SEM and are representative of 3 experiments run in triplicate.

## Abbreviations

BER: Base excision repair
DMSO: Dimethyl sulfoxide
EGF: Epidermal growth factor
ErbB: Epidermal growth factor receptor
GPCR: G protein-coupled receptor
HN2: Mechlorethamine
HRR: Homologous recombination repair
ICL: Interstrand crosslink
MAPK: Mitogen-activated protein kinase
NER: Nucleotide excision repair
NHEJ: Nonhomologous end joining
OCT: Octreotide
PI: Propidium iodide
PVDF: Polyvinylidene difluoride
RTK: Receptor tyrosine kinase
SST: Somatostatin
SSTR: Somatostatin receptor
TK: Tyrosine kinase
TKI: Tyrosine kinase inhibitor
TMZ: Temozolomide.
